# Epithelioid Angiosarcoma of the Small Intestine After Occupational Exposure to Radiation and Polyvinyl Chloride: A case Report and Review of Literature

**DOI:** 10.1080/13577140500389069

**Published:** 2005

**Authors:** Maged F. Khalil, Asha Thomas, Adel Aassad, Moshe Rubin, Robert N. Taub

**Affiliations:** 1 Division of Preventive Medicine & Nutrition Columbia University College of Physicians & Surgeons 435 Hillman Ave., Staten Island New York NY 10314 USA; 2 Department of Pathology Columbia University College of Physicians & Surgeons New York NY 10032 USA; 3 Division of Gastroenterology Columbia University College of Physicians & Surgeons New York NY 10032 USA; 4 Division of Medical Oncology Columbia University College of Physicians & Surgeons New York NY 10032 USA

## Abstract

Angiosarcomas represent 1–2% of soft tissue sarcomas and most frequently occur in the subcutis. They may affect internal organs, such as the heart, liver, and spleen, and only rarely do they emerge in the gastrointestinal tract. The association between angiosarcomas and certain toxic chemical substances or previous external-beam radiation therapy is well documented.


*Sarcoma*, September/December 2005; 9(3/4): 161–164

Taylor & Francis

Taylor & Francis Group

CASE REPORT

### Epithelioid angiosarcoma of the small intestine after occupational exposure to radiation and polyvinyl chloride: A case report and review of literature

MAGED F. KHALIL[1], ASHA THOMAS[1], ADEL AASSAD[2], MOSHE RUBIN[3], & ROBERT N. TAUB[4]

*Divisions of Preventive [1]Medicine & Nutrition, [3]Gastroenterology and [4]Medical Oncology and [2]Department of Pathology, Columbia University College of Physicians & Surgeons, New York, NY 10032, USA*

*(Received 7 March 2005)*

**Abstract**
Angiosarcomas represent 1–2% of soft tissue sarcomas and most frequently occur in the subcutis. They may affect internal organs, such as the heart, liver, and spleen, and only rarely do they emerge in the gastrointestinal tract. The association between angiosarcomas and certain toxic chemical substances or previous external-beam radiation therapy is well documented.

**Keywords:** *Abdominal pain, CD31, CD34, capsule endoscopy, angiosarcoma/etiology/diagnosis/pathology/surgery, intestinal neoplasms/diagnosis/pathology/surgery, intestine, small, neoplasms, radiation-induced*

### Introduction

The following case, the fourth report of an epithelioid variant of angiosarcoma of the ileum highlights the possible relation between this extremely rare tumor and occupational exposure to radiation and chemicals such as polyvinyl chloride.

### Report of a case

### *Clinical history*

This 68-year-old restaurateur, who used to work as a chemist with 30 years history of heavy occupational exposure to radiation and polyvinyl chloride developed GI bleeding and melena in January 2000. Esophagogastroduodenscopy and colonoscopy were both negative and the symptoms disappeared.

In April 2003 the patient developed right lower quadrant and epigastric recurrent crampy abdominal pain, associated with melena. He was evaluated with upper and lower endoscopies and then capsule endoscopy, which showed a probable mass at the ileocecal junction (Figure 1).

Abdominal and pelvic CT scan showed a $5 \times 4.5 \times 4.5$-cm right lower quadrant mass, which seemed to arise in the iliac wing of the right iliac fossa. It showed central low attenuation suggesting necrosis and stranding of fat. An isotopic scan for Meckel's diverticulum was inconclusive.

On 16 July 2003 the patient was taken to the operating room for laparoscopic-assisted small bowel resection and partial abdominal wall resection. At surgery he was found to have a loop of small bowel adherent to the right lower quadrant anterior and anterolateral abdominal wall just outside of the pelvis, well above the ureter and iliac and gonadal vessels. The mesenteric loop had a mass in it and the small bowel had a mass as well. The mass was resected together with an adherent portion of abdominal wall. He subsequently recovered from the surgery. The pathology report at that time showed a high-grade angiosarcoma with extensive necrosis, hemorrhage, and a mitotic rate of 18 mitoses per 10 high power fields (Figure 2d).

The sarcoma involved the full thickness of the bowel wall, extensively infiltrated mesenteric fat and infiltrated submucosa and mucosa causing broad ulceration, the growth pattern of the angiosarcoma resembled slit-like blood-filled spaces in some areas, larger vascular spaces with papillary intraluminal

Correspondence: M. F. Khalil, MD, 435 Hillman Ave., Staten Island, NY 10314, USA. Tel: 1 718 809 3852. Fax: 1 718 306 0916. E-mail: magedkhalil594@aol.com

ISSN 1357–714X print/ISSN 1369–1643 online/05/(03-04)0161-4 © 2005 Taylor & Francis
DOI: 10.1080/13577140500389069

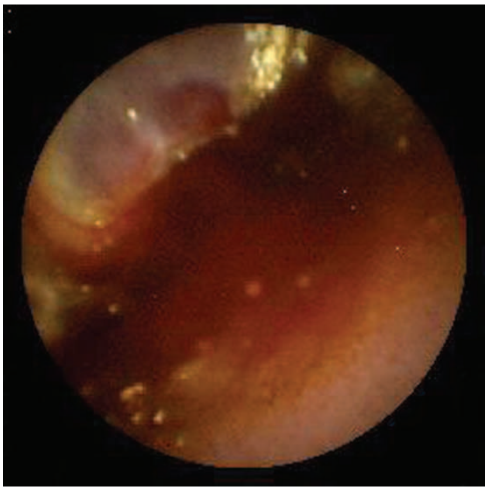

Figure 1. Capsule endoscopy image: protruding tumor with dark bloody fluid beyond it.

protrusions in other areas (Figure 2a–c). Tumor was focally present at the mesenteric line of resection. No tumor was seen at the proximal and distal lines of resection of the bowel. Five mesenteric lymph nodes were negative for metastasis. No pathological change was seen in random sections of ileum.

Immunohistochemical stains of tumor tissue (Figure 2e,f) showed a strong positive staining for the endothelial markers CD31 and CD34, confirming the angiosarcomatous nature of this sarcoma. There is no staining for the muscle marker desmin, S-100, cytokeratin or CD117.

A follow-up scan on 9 September 2003 showed a $1.5 \times 1.2$-cm spiculated lesion in the mesentery anterior to the bifurcation of the right iliac vessel.

A PET scan showed increased activity in the right side of the abdomen consistent with recurrent tumor.

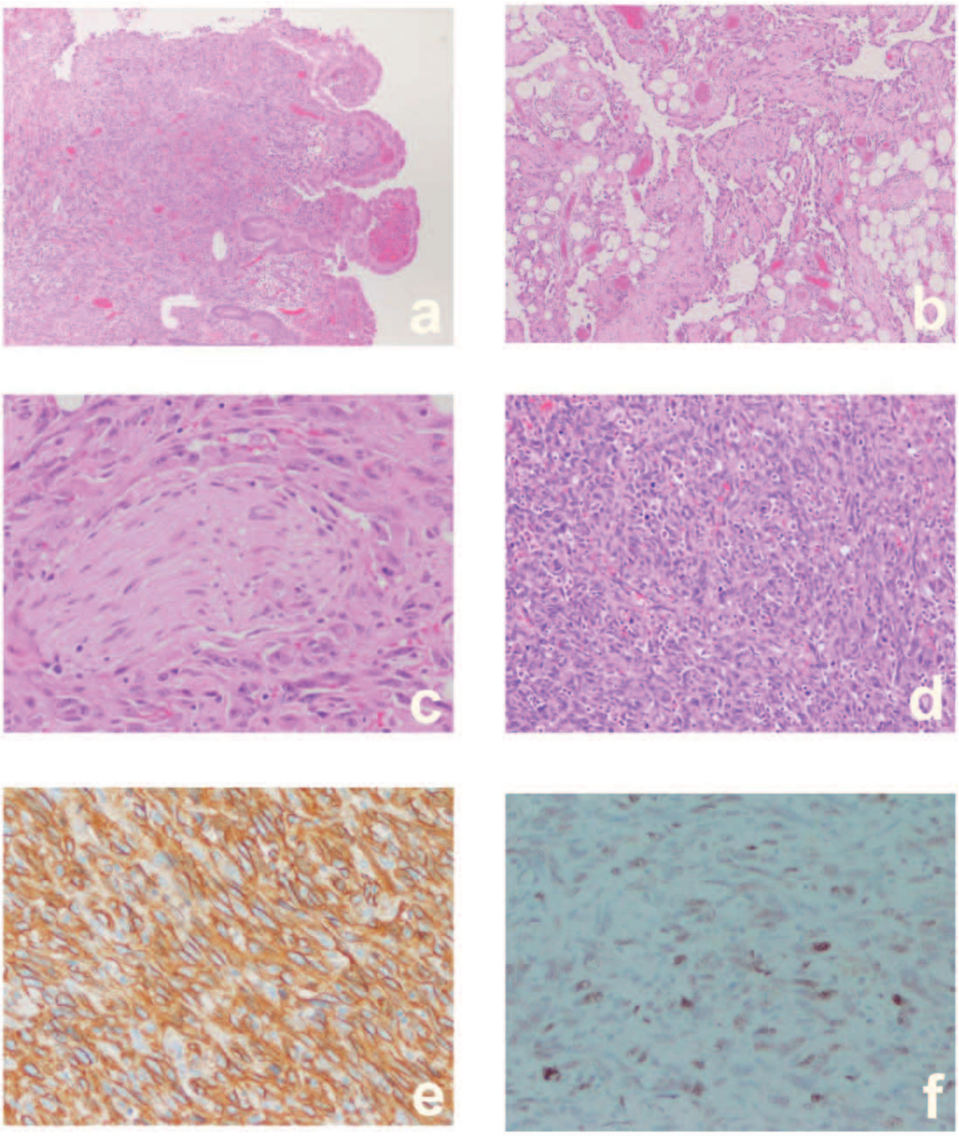

Figure 2. (a) Low power view shows an infiltrative neoplasm that has invaded through the muscle wall and reached the small intestinal mucosa. (b, c) The tumor forms inter-anastomosing channels that extensively infiltrate mesenteric adipose tissue and (c) around nerves. (d) High power view showing elongated tumor cells with hyperchromatic, pleomorphic nuclei and numerous mitotic figures. The tumor forms vascular spaces containing red blood cells. (e) CD31 immunohistochemistry shows strong, diffuse, membranous staining of the tumor cells. (f) Immunohistochemistry for p53 shows moderate to strong staining of the majority of tumor cell nuclei.

There was a focal area of increased PET activity anterior and medial to the right iliac vessels thought to represent spread to celiac nodes.

He was readmitted in October 2003 for rapidly increasing abdominal distention and ascites, necessitating urgent exploratory laparoscopy. At that time there was massive loculated ascites, which could not be drained, and considerable peritoneal studding. Frozen sections showed a poorly differentiated neoplasm. The tumor cells grew in sheets and epithelioid nests, and invaded diffusely into the fat of the abdominal wall. In some foci there were cleft-like spaces within nests of tumor cells; the lesion appeared more epithelioid with fewer well-formed vascular spaces. The tumor cells were strongly positive for vimentin and CD31.

After surgery he continued to be distended and uncomfortable, but was passing small amounts of gas. Chemotherapy with doxorubicin and ifosfamide was suggested. However, the patient developed severe respiratory distress, and expired before starting on chemotherapy.

### Discussion

Angiosarcomas are rare neoplasms characterized by proliferation of tumor cells with vascular endothelial features, accounting for only 1–2% of all soft tissue sarcomas [1,2]. They occur most commonly in the scalp, skin and soft tissues of the head and neck region in elderly men.

Intra-abdominal angiosarcomas are very rare neoplasms, which usually arise in the liver or spleen and extremely rarely in the gastrointestinal tract. From 1965 till 2003, only 13 cases of small intestine angiosarcomas have been reported [1,3–13] in the English literature.

Although the precise predisposing factors of this tumor remain unclear, exposure to vinyl chloride (VC), thorotrast, arsenic chemotherapy, trauma, long-standing lymphoedema, and radiotherapy have been implicated in its pathogenesis [1,2,14]

VC is a known chemical carcinogen known to cause angiosarcoma of the liver (ASL). Because ASL has a latency of approximately 20 years, mortality from ASL caused by VC exposure is still to be expected for some time, even if VC is no longer used industrially in the United States.

Parallel to human case reports, VC is carcinogenic in rats [15,16]. Extensive studies in the rat, mouse, and hamster on the effects of oral [17,18] and inhalation [19,20] exposure to VC have shown it to be both genotoxic and carcinogenic, causing a wide spectrum of tumors, including ASL and other liver tumors, mammary gland carcinoma, neuroblastoma, nephroblastoma, forestomach and lung tumors, and Zymbal gland tumor, in various animal species.

External-beam irradiation given either for malignancy (most commonly carcinoma of the cervix or ovary) or for benign lymphangioma has preceded several reported cases of abdominal angiosarcomas [7–9]. The dose of prior radiation ranged from 40–80 Gy, and the median time of development of the angiosarcoma was 12.5 years (range, 2.5–50 years) [5,21]. Postradiation angiosarcomas have been described in the omentum, small intestine, colon, and in the form of diffuse angiosarcomatosis of the entire abdomen [5,10,21].

In 1948, Stewart and Treves described the development of lymphangiosarcoma in a post-mastectomy edematous extremity. The median time from mastectomy to the development of lymphangiosarcoma is 10 years (range, 5–27 years) [22]. Although the etiology is unknown, lymph node dissection complicated by lymphoedema, trauma and irradiation are important risk factors [23–27].

In addition, foreign bodies were shown to be associated with angiosarcomas in intra-abdominal sites and in the colon [28,29].

Clinically, small intestinal angiosarcomas usually present with gastrointestinal bleeding and/or intestinal obstruction [1].

Because of the anatomical location of the small intestine, bleeding sites are often difficult to detect using conventional radiological or scintigraphic techniques, and this sometimes makes laparotomy or surgical intervention necessary [30,31]. Capsule endoscopy is emerging as a sensitive technique and it is now possible to visualize small-intestinal bleeding sites beyond the range of regular endoscopes [32].

The pathological diagnosis may be a challenge, and immunohistological markers are needed to differentiate the tumor from poorly differentiated carcinomas. Angiosarcomas commonly show a double growth pattern, vasoformative and/or solid. The vasoformative structures can range from well-formed vessels to vascular spaces with papillary projections to slit-like anastomosing vascular channels. These neoplastic vessels are lined by spindle or plump anaplastic endothelial cells with slight or moderate nuclear pleomorphism and multilayering. The solid growth pattern consists of two cell types: sheets of spindle-shaped cells or large, polygonal epithelioid cells with abundant amphophilic or eosinophilic cytoplasm, with moderate to marked nuclear pleomorphism, vesicular nuclei, and large central nucleoli and occasional intracytoplasmic vacuolization. The mitotic rate is high.

Immunohistochemical staining with expression of endothelial markers (factor VIII-related antigen, CD31, CD34) is needed for definitive diagnosis [33,34].

Before this report, only three cases of an intestinal epithelioid angiosarcoma have been previously described, two of which were multifocal [3,9].

In summary, even though angiosarcomas of the small intestine are rare, such a diagnosis might be a consideration in elderly patients with significant vinyl chloride exposure and characteristic symptoms. As in this case, capsule endoscopy and PET scan can contribute to the diagnosis, which can be made definitively by pathological and Immunohistochemical examination.

### References

1. Chami TN, Ratner LE, Henneberry J. Angiosarcoma of the small intestine: A case report and literature review. Am J Gastroenterol 1994;89(5):797–800.
2. Naka N, Ohsawa M, Tomita Y. Angiosarcoma in Japan. A review of 99 cases. Cancer 1995;75(4):989–996.
3. Delvaux V, Sclot R, Neuville B. Multifocal epithelioid angiosarcoma of the small intestine. Virchows Arch 2000;437(1):90–94.
4. Knop FK, Hansen MB, Meisner S. Small-bowel hemangiosarcoma and capsule endoscopy. Endoscopy 2003;35(7):637.
5. Nanus DM, Kelsen D, Clark D.G. Radiation-induced angiosarcoma. Cancer 1987;60(4):777–779.
6. Chen KT, Hoffman KD, Hendricks EJ. Angiosarcoma following therapeutic irradiation. Cancer 1979;44(6): 2044–2048.
7. Ordonez NG, del Junco GW, Ayala A. Angiosarcoma of the small intestine: An immunoperoxidase study. Am J Gastroenterol 1983; 78(4):218–221.
8. Taxy JB, Battifora H. Angiosarcoma of the gastrointestinal tract. A report of three cases. Cancer 1988;62(1):210–216.
9. Watanabe K, Hoshi N, Suzuki T. Epithelioid angiosarcoma of the intestinal tract with endothelin-1-like immunoreactivity. Virchows Arch A Pathol Anat Histopathol 1993;423(4): 309–314.
10. Wolov RB, Sato N, Azumi N, Lack EE. Intra-abdominal 'angiosarcomatosis' report of two cases after pelvic irradiation. Cancer 1991;67(9):2275–2279.
11. Fraiman G, Ganti AK, Potti A. Angiosarcoma of the small intestine: A possible role for thalidomide? Med Oncol 2003;20(4): 397–402.
12. Kelemen K, Yu QQ, Howard L. Small intestinal angiosarcoma leading to perforation and acute abdomen: A case report and review of the literature. Arch Pathol Lab Med 2004;128(1):95–98.
13. Lourenco D, Vilaca D, Gomes A. Small intestine angiosarcoma associated with a foreign body. Ann Chir 2000;125(8):802–803.
14. Suzuki F, Salto A, Ishi K. Intra-abdominal angiosarcomatosis after radiotherapy. J Gastroenterol Hepatol 1999; 14(3):289–292.
15. Viola PL, Bigotti A, Caputo A. Oncogenic response of rat skin, lungs, and bones to vinyl chloride. Cancer Res 1971;31(5):516–522.
16. Viola, P.L, Pathology of vinyl chloride. Med Lav 1970. 61(3):147–80.
17. Til HP, Feron VJ, Immel H.R. Lifetime (149-week) oral carcinogenicity study of vinyl chloride in rats. Food Chem Toxicol 1991;29(10):713–718.
18. Feron VJ, Hendriksen CF, Speek AJ. Lifespan oral toxicity study of vinyl chloride in rats. Food Cosmet Toxicol 1981;19(3):317–333.
19. Maltoni C, Lefemine G, Ciliberti A. Carcinogenicity bioassays of vinyl chloride monomer: A model of risk assessment on an experimental basis. Environ Health Perspect 1981;41:3–29.
20. Lee CC, Bhandari JC, Winston JM. Carcinogenicity of vinyl chloride and vinylidene chloride. J Toxicol Environ Health 1978;4(1):15–30.
21. Westenberg AH, Wiggers T, Henzen-Logmans SC. Post-irradiation angiosarcoma of the greater omentum. Eur J Surg Oncol 1989;15(2):175–178.
22. Kaufmann T, Chu F, Kaufman R. Post-mastectomy lymphangiosarcoma (Stewart-Treves syndrome): Report of two long-term survivals. Br J Radiol 1991;64(765):857–860.
23. Borel Rinkes IH, de Jongste AB. Lymphangiosarcoma in chronic lymphedema. Reports of 3 cases and review of the literature. Acta Chir Scand 1986;152:227–230.
24. D'Amore ES, Wick MR, Geisinger KR. Primary malignant lymphoma arising in postmastectomy lymphedema. Another facet of the Stewart-Treves syndrome. Am J Surg Pathol 1990;14(5): 456–463.
25. Foldi E, Foldi M, Weissleder H. Conservative treatment of lymphoedema of the limbs. Angiology 1985;36(3):171–180.
26. Hultberg BM. Angiosarcomas in chronically lymphedematous extremities. Two cases of Stewart-Treves syndrome. Am J Dermatopathol 1987;9(5):406–412.
27. Noguchi M, Hasegawa H, Tajiri K. Stewart-Treves syndrome. A report of two cases with a review of Japanese literature. Jpn J Surg 1987;17(5):407–412.
28. Ben-Izhak O, Kerner H, Brenner B. Angiosarcoma of the colon developing in a capsule of a foreign body. Report of a case with associated hemorrhagic diathesis. Am J Clin Pathol 1992;97(3):416–420.
29. Jennings TA, Peterson L, Axiotis CA. Angiosarcoma associated with foreign body material. A report of three cases. Cancer 1988;62(11):2436–2444.
30. Lingenfelser T, Ell C. Lower intestinal bleeding. Best Pract Res Clin Gastroenterol 2001;15(1):135–153.
31. Costamagna G, Shah SK, Tringali A. Prospective evaluation of a new self-expanding plastic stent for inoperable esophageal strictures. Surg Endosc 2003;17(6):891–895.
32. Lewis BS, Swain P. Capsule endoscopy in the evaluation of patients with suspected small intestinal bleeding: Results of a pilot study. Gastrointest Endosc 2002;56(3):349–53.
33. Alles JU, Bosslet K. Immunocytochemistry of angiosarcomas. A study of 19 cases with special emphasis on the applicability of endothelial cell specific markers to routinely prepared tissues. Am J Clin Pathol 1988;89(4):463–471.
34. Burgdorf WH, Mukai K, Rosai J. Immunohistochemical identification of factor VIII-related antigen in endothelial cells of cutaneous lesions of alleged vascular nature. Am J Clin Pathol 1981;75(2):167–171.

